# An Unusual Case of Cirrhosis

**DOI:** 10.1155/2014/670176

**Published:** 2014-02-04

**Authors:** Ahmad Alkaddour, Kenneth J. Vega, Adil Shujaat

**Affiliations:** ^1^Department of Medicine, University of Florida College of Medicine Jacksonville, 655 West 8th Street, Jacksonville, FL 32209, USA; ^2^Department of Medicine, University of Oklahoma Health Sciences Center, Oklahoma City, OK 73104, USA; ^3^Division of Pulmonary, Critical Care and Sleep Medicine, University of Florida College of Medicine-Jacksonville, 655 West 8th Street, Jacksonville, FL 32209, USA

## Abstract

49-year-old white female with remote h/o sarcoidosis was referred to GI when her liver was noted to be nodular. Physical examination revealed normal vital signs and no icterus, spider nevi, clubbing, ascites, hepatosplenomegaly, or ankle edema. LFTs, hepatitis serologies, ANA, AMA, ASMA, Ferritin, Ceruloplasmin, and **α**1-AT, level were unremarkable. Liver biopsy showed cirrhosis. She developed worsening of baseline SOB and was hospitalized. She was eventually diagnosed with constrictive pericarditis. A diagnosis of cardiac cirrhosis was made.

## 1. Introduction

Constrictive pericarditis is a rare but severely disabling consequence of the chronic inflammation of the pericardium, leading to an impaired filling of the ventricles and reduced ventricular function [1]. The timely diagnosis of a cardiac etiology of liver dysfunction is important because such dysfunction is potentially reversible if the underlying cardiac disease is treated before the development of frank cirrhosis [2, 3].

Below, we present a case of a 49-year-old female who was incidentally found to have cirrhosis. Initial workup was negative. Thoracic imaging showed pericardial calcifications which ultimately led to the diagnosis of constrictive pericarditis. We will briefly discuss the literature on cardiac causes of liver cirrhosis.

## 2. Case Report

49-year-oold white female with remote h/o sarcoidosis was referred to GI when her liver was noted to be nodular during laparoscopy for an ovarian cyst. She denied fatigue, vomiting-up blood, abdominal distension and pain, ankle swelling, itching, yellow discoloration of skin and eyes, and episodes of confusion or sleepiness. She denied alcohol abuse. Physical examination revealed normal vital signs and no icterus, spider nevi, clubbing, ascites, hepatosplenomegaly, or ankle edema. LFTs revealed mild elevation in alkaline phosphatase and PT was slightly prolonged. CBC showed mild thrombocytopenia. Hepatitis serologies, ANA, AMA, ASMA, Ferritin, Ceruloplasmin, and *α*1-AT, level were unremarkable. A liver biopsy was done. It confirmed cirrhosis. Biopsy did not show any granulomas but showed sinusoidal dilatation which prompted a referral to cardiology. ECHO showed enlarged IVC and was otherwise unremarkable. A left and right heart catheterization was done. LHC showed normal coronaries and RHC showed RAP of 12 mm Hg, PAP of 32/15 (mean 21) mm Hg, PAWP of 18 mm Hg, LVEDP of 18 mm Hg, and CO of 5.2 L/min. She developed worsening shortness of breath and was referred to pulmonary medicine. PFTs showed mild restriction but CXR was unrevealing. CTPA ruled out PE and showed scattered pericardial calcification. Bubble ECHO did not show a right to left shunt. She developed SVT and was hospitalized. She underwent a repeat left and right heart catheterization: RAP was 25 mm Hg, PAP 52/25 (mean 37) mm Hg, PAWP 32 mm Hg, LVEDP 36 mm Hg, and CO 4.12 L/min. Simultaneous measurement of left- and right-sided pressures confirmed constrictive pericarditis. In retrospect, the cirrhosis is “cardiac” cirrhosis and the result of long-standing elevated right-sided heart pressures. Similarly her dyspnea on exertion can be explained by constrictive pericarditis. We believe that constrictive pericarditis resulted from sarcoidosis. She is now being evaluated for pericardiectomy.

## 3. Discussion

Our case illustrates the importance of considering a cardiac etiology in the work-up of cirrhosis especially when the most common causes are not found. Classically, serum albumin is normal unless frank cirrhosis has developed; ascitic fluid analysis reveals an elevated serum albumin-ascites gradient (>1.1 g/dL) typical of portal hypertension and demonstrates an elevated total protein level (>2.5 g/dL) [4]. In our case, the LFTs were only mildly abnormal with a mildly elevated PT and mildly elevated alkaline phosphatase. The finding of a nodular liver was incidental and no primary liver disease was found. Sinusoidal dilation indicating congestive hepatopathy helped steer us towards a cardiac etiology; other findings on liver biopsy that may be found include sinusoidal degeneration, variable degrees of hemorrhagic necrosis in zone 3, fatty change, and variable degrees of cholestasis [4]. In general, patients with passive hepatic congestion do not have stigmata of portal hypertension such as spider angiomata or evidence of portosystemic shunts such as caput medusa [4]. Patients with constrictive pericarditis, however, may develop ascites. Nevertheless, such findings were absent in our patient.

Constrictive pericarditis is a rare but severely disabling consequence of the chronic inflammation of the pericardium, leading to an impaired filling of the ventricles and reduced ventricular function [1].

Establishing the diagnosis of constrictive pericarditis and secondary congestive hepatopathy/cirrhosis remains a challenge. One study reported a median delay in diagnosis of greater than 10 years [5]. In another case, the diagnosis of constrictive pericarditis was made only after liver transplantation was performed for presumed cryptogenic cirrhosis [6]. Much of the difficulty in diagnosing constrictive pericarditis can be attributed to its insidious course and the fact that some of the symptoms and signs resulting from it, that is, dyspnea and ascites, can be mistakenly thought to result from primary liver dysfunction. Dyspnea in a patient with liver dysfunction usually makes one suspects hepatopulmonary syndrome, portopulmonary hypertension, cardiomyopathy, hepatic hydrothorax, ascites, or anemia, whereas ascites itself is a hallmark of liver disease complicated by portal hypertension. Constrictive pericarditis is curable and should be considered in all cases of unexplained cirrhosis, regardless of atypical hepatic histology [7].

**Figure 1:**
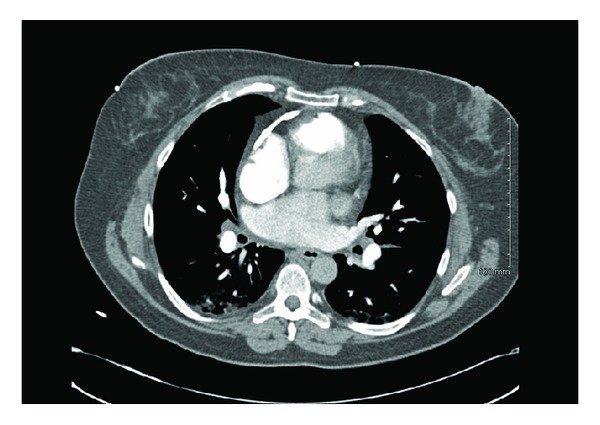
CT scan of chest showing pericardial calcifications.

Pericardial involvement is uncommon in sarcoidosis even in the presence of extensive myocardial infiltration. It is observed in fewer than 10% of patients with cardiac sarcoidosis [8]. Pericardial calcification should provide a clue to the diagnosis of constrictive pericarditis (see Figure 1). One case study demonstrated the importance of this finding on low-cost imaging to help point towards this diagnosis [9]. The major differential of constrictive pericarditis is restrictive cardiomyopathy which is more common in patients with sarcoidosis. Although echocardiography and cardiac CT/MR might distinguish one from the other, the most specific finding differentiating constrictive pericarditis from restrictive cardiomyopathy is demonstrated on heart catheterization. Simultaneous measurement of the left and right heart pressures demonstrates respiratory variation in ventricular filling and increased ventricular interdependence along with increased atrial pressures and equalization of end-diastolic pressures [10].

The timely diagnosis of a cardiac etiology of liver dysfunction is important because such dysfunction is potentially reversible if the underlying cardiac disease is treated before the development of frank cirrhosis [2, 3]. Moreover, early treatment of underlying cardiac disease might also prevent the development of hepatocellular carcinoma as suggested by an interesting case study in which a patient with negative hepatitis serologies and cirrhosis secondary to constrictive pericarditis developed hepatocellular carcinoma confirmed by biopsy [11].

## 4. Conclusion

This case study illustrates to gastroenterologists the need to consider a cardiac etiology in the work-up of cirrhosis especially when the most common causes are not found and if liver biopsy shows evidence of congestive hepatopathy and is inconclusive for primary liver disease. The presence of pericardial calcification in a patient with cirrhosis should suggest the possibility of constrictive pericarditis and cardiac cirrhosis and prompt cardiac MR/CT imaging and cardiac catheterization. Simultaneous invasive measurement of the pressures in the right and left heart chambers by an experienced cardiologist is the key to making the diagnosis. Constrictive pericarditis is a rare but well-known manifestation of sarcoidosis. Prompt referral to a thoracic surgeon for pericardiectomy is important once the diagnosis is made to prevent progression to cirrhosis.
